# Gallium Uncouples Iron Metabolism to Enhance Glioblastoma Radiosensitivity

**DOI:** 10.3390/ijms251810047

**Published:** 2024-09-18

**Authors:** Stephenson B. Owusu, Amira Zaher, Stephen Ahenkorah, Darpah N. Pandya, Thaddeus J. Wadas, Michael S. Petronek

**Affiliations:** 1 Department of Radiation Oncology, Division of Free Radical and Radiation Biology, The University of Iowa, Iowa City, IA 52242, USA; stephensonboakye-owusu@uiowa.edu (S.B.O.); amira-zaher@uiowa.edu (A.Z.); 2 Department of Radiology, The University of Iowa, Iowa City, IA 52242, USA; stephen-ahenkorah@uiowa.edu (S.A.);

**Keywords:** glioblastoma, gallium nitrate, radiosensitivity, mitochondrial iron metabolism

## Abstract

Gallium-based therapy has been considered a potentially effective cancer therapy for decades and has recently re-emerged as a novel therapeutic strategy for the management of glioblastoma tumors. Gallium targets the iron-dependent phenotype associated with aggressive tumors by mimicking iron in circulation and gaining intracellular access through transferrin-receptor-mediated endocytosis. Mechanistically, it is believed that gallium inhibits critical iron-dependent enzymes like ribonucleotide reductase and NADH dehydrogenase (electron transport chain complex I) by replacing iron and removing the ability to transfer electrons through the protein secondary structure. However, information regarding the effects of gallium on cellular iron metabolism is limited. As mitochondrial iron metabolism serves as a central hub of the iron metabolic network, the goal of this study was to investigate the effects of gallium on mitochondrial iron metabolism in glioblastoma cells. Here, it has been discovered that gallium nitrate can induce mitochondrial iron depletion, which is associated with the induction of DNA damage. Moreover, the generation of gallium-resistant cell lines reveals a highly unstable phenotype characterized by impaired colony formation associated with a significant decrease in mitochondrial iron content and loss of the mitochondrial iron uptake transporter, mitoferrin-1. Moreover, gallium-resistant cell lines are significantly more sensitive to radiation and have an impaired ability to repair any sublethal damage and to survive potentially lethal radiation damage when left for 24 h following radiation. These results support the hypothesis that gallium can disrupt mitochondrial iron metabolism and serve as a potential radiosensitizer.

## 1. Introduction

Gallium-based therapeutic strategies are thought to be an effective means to promote tumor cell killing and have been studied in this arena for decades. The mechanism of gallium-based therapy is believed to be primarily due to the ability of gallium (more specifically, Ga^3+^ ions) to mimic iron ions while removing the redox component as gallium exhibits similar iron binding despite its inert nature. Ga(NO_3_)_3_ is known to gain entry to cells by mimicking iron, binding transferrin receptors (TfRs), and being internalized by TfR-mediated endocytosis [1,2,3]. Recently, gallium maltolate (GaM) has shown significant therapeutic potential in the management of glioblastoma (GBM) tumors [4,5]. Moreover, gallium toxicity has also been linked to the induction of apoptosis [6,7]. Mechanistically, it has been purported that gallium-induced cell death is due to its ability to inhibit ribonucleotide reductase and impair NADH dehydrogenase (electron transport chain complex I) activity [4,8,9]. Ribonucleotide reductase is a di-ferric enzyme that catalyzes the de novo conversion of nucleotides to deoxyribonucleotides for effective DNA synthesis [10]. Conversely, NADH dehydrogenase (electron transport chain complex I) is an Fe-S cluster-containing enzyme where the formation of the Fe-S cluster is required for enzymatic activity [11,12,13]. Moreover, gallium has been shown to inhibit Fe-S synthesis on the iron–sulfur cluster assembly enzyme, ISCU, which is the scaffold protein for Fe-S biogenesis [4,14]. Taken together, these observations suggest that gallium may have more global effects on iron metabolism associated with the disruption of mitochondrial iron homeostasis. Mitochondrial iron metabolism is intimately connected to DNA metabolism through Fe-S biogenesis, which provides the necessary substrate to numerous DNA metabolic proteins (e.g., DNA polymerases, helicases, glycosylases, etc.) in the form of [4Fe-4S]^2+^ clusters [15]. Moreover, enriched Fe-S biogenesis activation has been proposed to support tumor growth [14]. Beyond specific protein inhibition, the effects of gallium on mitochondrial iron metabolism have yet to be studied. Thus, an alternative hypothesis to the current understanding of gallium toxicity is that gallium generally impairs mitochondrial iron metabolism to promote genomic instability. The goal of this study is to test the implications of gallium nitrate (Ga(NO_3_)_3_ on mitochondrial iron metabolic disruptions in GBM cells.

## 2. Results

### 2.1. Ga(NO_3_)_3_ Induces an Fe Depletion Phenotype in Glioblastoma Cells 

**Figure 1:**
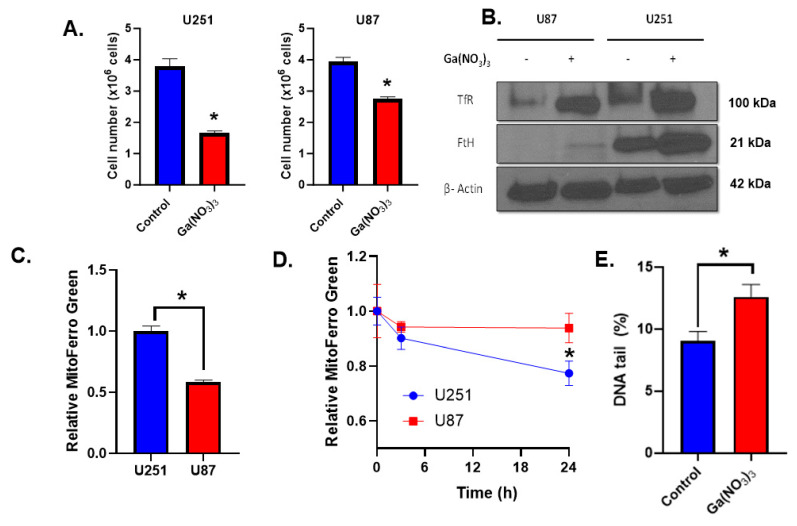
Ga(NO_3_)_3_ causes mitochondrial iron depletion associated with DNA damage induction. (**A**) Cell counts in U251 and U87 GBM cells following a 48 h, 500 µM Ga(NO_3_)_3_ treatment. Error bars represent mean ± SD of three measures with * *p* < 0.05 using Welch’s *t*-test. (**B**) Western blot analysis of transferrin receptor (TfR) and ferritin heavy chain (FtH) expression following a 24 h, 500 µM Ga(NO_3_)_3_ treatment. β-actin is used as a loading control. (**C**) Baseline mitochondrial iron content in U251 and U87 GBM cells (normalized to U251 cells). Error bars represent mean ± SD of three measures with * *p* < 0.05 using Welch’s *t*-test. (**D**) Temporal effects of 500 µM Ga(NO_3_)_3_ on mitochondrial iron content measured at 3 and 24 h. Error bars represent mean ± SD of three measures with * *p* < 0.05 using a two-way ANOVA test. (**E**) Single-stranded DNA damage analyzed using an alkaline comet assay in U251 cells following a 24 h treatment of Ga(NO_3_)_3_. Error bars represent mean ± SD of three measures with * *p* < 0.05 using Welch’s *t*-test.

Initially, the effects of Ga(NO_3_)_3_ on iron metabolism were evaluated. A 500 µM dose was chosen for this study based on previous studies, which reported cytotoxic doses of Ga(NO_3_)_3_ in the range of 100–500 µM [16]. When treated with 500 µM Ga(NO_3_)_3_ for 48 h, a significant growth delay was observed in both U251 and U87 glioblastoma cells (Figure 1A). Because gallium can mimic iron binding, toxicities associated with Ga(NO_3_)_3_ overload have been hypothesized to be linked to the displacement of iron from enzymes, which would presumably result in increases in freely chelatable (i.e., labile) iron. When cells were lysed and analyzed for total labile iron, minimal changes in labile iron per cell were observed; however, each cell line did show small increases that only trended towards significance in the U87 cells (Figure S1A). The acute effects of Ga(NO_3_)_3_ on labile iron were further investigated using a flow-cytometry-based probe, Calcein-AM. Consistent with the changes in labile iron observed after 48 h, there was a significant increase in labile iron in U87 cells, while U251 cells remained largely unaffected, despite the increased sensitivity (Figure S1B). Therefore, while Ga(NO_3_)_3_ inhibits cell growth in both glioblastoma cell lines, it appears that this effect does not correlate with the displacement of iron within the cell in the short or long term. To further investigate this effect, FtH was overexpressed in the U251 cells to evaluate if increasing their iron storage capacity can mediate Ga(NO_3_)_3_ toxicity. However, ferritin heavy chain overexpression was unable to mitigate Ga(NO_3_)_3_ toxicity (*p* = 0.44, Figure S2). Thus, it appears that Ga(NO_3_)_3_ toxicity is not associated with the labile iron pool or iron-catalyzed reactions like those associated with ferroptosis induction.

Because the observed toxicity associated with Ga(NO_3_)_3_ does not appear to correlate with changes in labile iron content, the impact on global iron metabolic features was investigated. Following a 24 h treatment with 500 µM Ga(NO_3_)_3_, both U251 and U87 cells exhibit robust enhancement of transferrin receptor (TfR) and ferritin heavy chain (FtH) protein expression (Figure 1B). This is consistent with a phenotypic change associated with iron depletion as both proteins are central to the maintenance of iron homeostasis through the uptake and storage of iron, respectively [17,18]. The robust changes in TfR expression caused by Ga(NO_3_)_3_ in both cell lines led to an investigation of the effects of transferrin supplementation on Ga(NO_3_)_3_ toxicity. Interestingly, the addition of 100 µg mL^−1^ of holo-transferrin as a cell culture supplement did not affect the toxicity associated with a 24 h treatment of Ga(NO_3_)_3_ in either cell line (Figure S3). Taken together, these results suggest that Ga(NO_3_)_3_ may induce an internal Fe depletion phenotype that may be more closely related to its cause of cell death; however, the gallium still exhibits cytostatic and cytotoxic effects in U87 cells where there was an increase in labile iron, which suggests that iron catalyzed oxidative damage may play a mechanistic role as has been previously hypothesized and may warrant further consideration [19]. 

Mitochondria house the Fe-S biogenesis and heme synthesis pathways, and thus, mitochondrial Fe content can influence global iron metabolism. As iron response proteins functionally depend on Fe-S clusters, mitochondrial iron status can have broader effects on iron homeostasis [20,21]. The trafficking of Fe to the mitochondria can be facilitated by endocytosis of the Tf-TfR complex, which can be modulated by Ga(NO_3_)_3_. Therefore, it was hypothesized that Ga(NO_3_)_3_ disrupts mitochondrial iron [22]. Based on the initial observation that labile iron was increased in only U87 cells, mitochondrial Fe content was characterized in these cells. At basal levels, U251 cells have significantly greater (≈2-fold) mitochondrial iron content than U87 cells (Figure 1C), suggesting an increased dependence on mitochondrial Fe metabolism. Following acute exposure to Ga(NO_3_)_3_ (3 h, 500 µM), there was a decrease in U251 mitochondrial Fe (*p* = 0.06), which opposed the enhancement by ferrous ammonium sulfate (Figure 1D and Figure S4). No changes were observed in the U87 cells (*p* = 0.96). Moreover, this effect was exacerbated following a 24 h treatment (500 µM) in U251 cells (*p* < 0.05, Figure 1D). Additionally, a trend towards mitochondrial iron depletion was observed in U87 cells after 24 h (*p* = 0.38). Therefore, these data support the hypothesis that the effects of Ga(NO_3_)_3_ on iron metabolism may associated with the depletion of mitochondrial iron. 

To further interrogate this effect, U251 cells were utilized due to their apparent mitochondrial iron dependence relative to the U87 cells. Furthermore, a 24 h treatment of Ga(NO_3_)_3_ significantly enhanced single-stranded DNA damage in U251 cells, consistent with the notion that mitochondrial iron metabolism serves as a critical connection between iron and DNA metabolism [15]. Therefore, it appears that Ga(NO_3_)_3_ can cause mitochondrial iron depletion that may, in turn, induce DNA damage in glioblastoma cells. 

### 2.2. Gallium-Resistant Cells Are Unstable and Have Impaired Mitochondrial Iron Uptake

**Figure 2:**
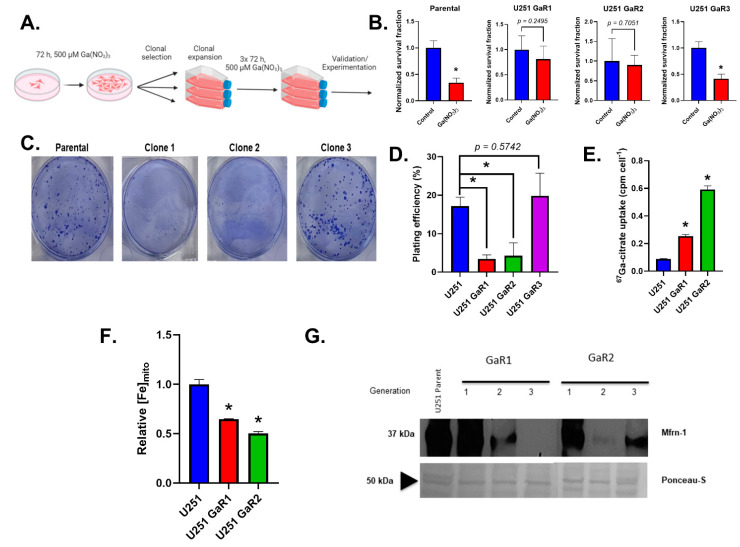
Gallium-resistant cells are highly unstable and have impaired mitochondrial iron uptake. (**A**) Gallium-resistant U251 cell lines were generated via three separate clonal selections following a 72 h, 500 µM treatment of Ga(NO_3_)_3_ for generating stable cell lines. (**B**) Ga(NO_3_)_3_ resistance was confirmed using a clonogenic survival assay with three separate clonally selected cell lines (GaR1,2,3) and parental U251 cells being treated with a 500 µM treatment of Ga(NO_3_)_3_ for 72 h. Error bars represent mean ± SEM (n = 3) with * *p* < 0.05 using Welch’s *t*-test. (**C**,**D**) Clonally selected cell lines (GaR1,2,3) with confirmed resistance to Ga(NO_3_)_3_ exhibit noticeable decreases in colony formation when plated as single cells (500 cells, **C**). This translates to a significant decrease in plating efficiency where plating efficiency (%) = # colonies counted/# cells plated (**D**). To combat this experimental difference, GaR1/2 were plated at higher concentrations (1000–2000 cells) for subsequent studies. Error bars represent mean ± SEM (n = 3) with * *p* < 0.05 using a one-way ANOVA with a post hoc Tukey’s test for multiple comparisons. (**E**) Parental U251, GaR1, and GaR2 cells were incubated with 200 µCi ^67^Ga-citrate for 2 h prior to harvesting, lysing, and analysis with a gamma counter to evaluate gallium uptake. (**F**) Analysis of basal mitochondrial iron content using MitoFerroGreen flow cytometry in Parental U251, GaR1, and GaR2 cells. Error bars represent mean ± SEM (n = 3) with * *p* < 0.05 using a one-way ANOVA with a post hoc Tukey’s test for multiple comparisons. (**G**) Western blot analysis of mitoferrin-1 expression (Mfrn-1) in the 3 separate generations of GaR1 and GaR2 cells following each individual round of clonal selection compared to parental U251 cells show an evolutionary loss of Mfrn-1.

Based on the preliminary observations that Ga(NO_3_)_3_ can induce a mitochondrial iron depletion to promote DNA damage in U251 cells, this cell line was exposed to long-term, high-dose Ga(NO_3_)_3_ in an attempt to generate gallium-resistant cell lines. To do this, the cells were treated with 500 µM Ga(NO_3_)_3_ for 72 h, and clones were allowed to form from single cells. Individual colonies were selected and expanded, with this process being repeated three times to generate stable cell lines (Figure 2A). Three separate clones were selected to evaluate Ga(NO_3_)_3_ sensitivity, where it was observed that two out of three clones were resistant to a subsequent 72 h, 500 µM Ga(NO_3_)_3_ dose (Figure 2B). During this validation, it was observed that the two resistant cell lines (GaR1 and GaR2) exhibited a significant decrease in the ability to form colonies, as evidenced by a significant decrease in plating efficiency (Figure 2C). Interestingly, both GaR1 and GaR2 show a significant increase in Ga-67 uptake (Figure 2D), which may signify a greater propensity for gallium and/or iron uptake and is consistent with the previously observed increase in TfR expression by Ga(NO_3_)_3_. Conversely, both GaR1 and GaR2 have significantly lower levels of mitochondrial iron (Figure 2E). Moreover, both GaR1 and GaR2 show a decrease in Mfrn-1 expression during the clonal selection process (Figure 2G), indicating that Ga(NO_3_)_3_ can cause an adaptive loss of Mfrn-1. Therefore, these data suggest that through the acquisition of Ga(NO_3_)_3_ resistance, there is an iron metabolic divergence associated with impaired mitochondrial iron intake.

### 2.3. Gallium-Resistant Cells Have Impaired Radiation Responses

**Figure 3:**
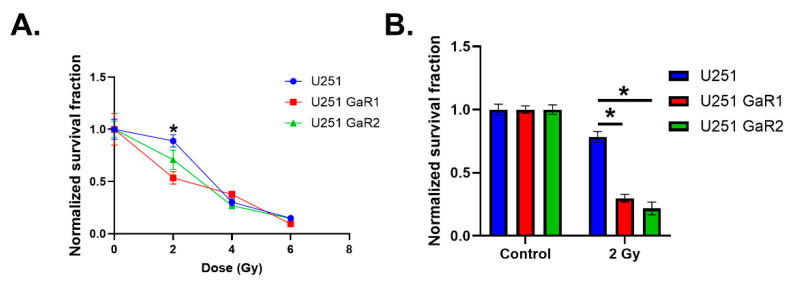
Mitochondrial iron metabolism modulates cell radiosensitivity. (**A**) Clonogenic survival analysis of radiation dose-dependent cell killing in parental U251, GaR1, and GaR2 cell lines. (**B**) Clonogenic survival analysis of U251, GaR1, and GaR2 cell lines treated with 2 Gy radiation and left for 24 h prior to plating. Error bars represent mean ± SEM (n = 3) with * *p* < 0.05 using a two-way ANOVA test with a post hoc test for multiple comparisons.

Due to the high level of instability observed in GaR1 and GaR2 cell lines compared to the parental U251 cells, it was hypothesized that they have increased sensitivity to ionizing radiation. Consistent with this hypothesis, GaR1 and GaR2 showed significantly greater radiosensitivity but only at 2 Gy, whereas this effect was lost at higher doses of radiation (Figure 3A). From a radiobiological perspective, these data suggest that GaR1 and GaR2 have a deficiency in sublethal damage repair, which would be consistent with the induction of single-stranded DNA damage caused by a single, 500 µM dose of Ga(NO_3_)_3_. Thus, cells were irradiated with 2 Gy and left for 24 h to allow for potentially lethal damage repair. This experiment revealed that when left unattended for 24 h following irradiation, GaR1 and GaR2 experienced significant cell death relative to the parental U251 cells (Figure 3B). Taken together, these data suggest that mitochondrial iron uptake may play an important role in cellular radiation responses. 

## 3. Discussion

Gallium-based therapeutics have recently re-emerged as an attractive strategy to enhance glioblastoma patient responses [4,5]. Fundamentally, gallium acts as an iron mimic to be internalized through TfR-mediated endocytosis [1,2,3]. The current mechanistic understanding with respect to gallium therapy is its ability to disrupt the function of iron-containing enzymes, namely, ETC complex I and ribonucleotide reductase [4,8,9]. Based on the results obtained in this current study, the effects of gallium may be more closely associated with a general disruption of iron metabolism characterized by mitochondrial iron depletion and the induction of DNA damage. The induction of DNA damage is convergent with its ability to inhibit ribonucleotide reductase [8,9], which is a phenomenon that is closely linked to impaired DNA damage repair [23]. Moreover, the observed mitochondrial iron depletion may also explain the inhibition of ribonucleotide reductase, as the formation of its di-ferric center is dependent on glutaredoxin-3 (GLRX3) of the iron–sulfur cluster biogenesis pathway [24,25]. This dependence on GLRX3 suggests that the iron used for the di-ferric center must first be chaperoned through the mitochondria. Thus, gallium may serve as a ribonucleotide reductase inhibitor, but the mechanism of action may likely be more closely linked to substrate limitation (i.e., mitochondrial iron depletion) as opposed or in addition to a direct inhibition; however, further mechanistic studies will be required to elucidate the role of each potential mechanism of action more completely. 

An important issue that has yet to be addressed is the mechanism via which gallium depletes mitochondrial iron. This can likely be linked to its redox inactivity. Because gallium is taken into cells through TfR-mediated endocytosis, it must first enter the endosomal iron trafficking system, a key first step for cellular iron delivery. A major limiting feature for the release of iron from the endosome into the cell is a key reduction step where, following the disassociation of the (Fe^3+^)_2_-Tf-TfR complex due to the low pH of the endosome, the free Fe^3+^ ions are reduced by the metalloreductase STEAP3 [26,27]. This step is essential because only Fe^2+^ ions can be transported out of the endosome through divalent-metal transporter 1 (DMT1) [28,29]. However, gallium ions (Ga^3+^; Ga(NO_3_)_3_) have a fully filled d-orbital valence shell and are unable to be reduced or oxidized, suggesting that gallium may enter the endosome but may not be able to be released [14]. As TfR-mediated iron delivery is a major feature that drives iron homeostasis globally, this effect would likely lead to a wholesale iron depletion phenotype, like the effects that have been observed in this study. Moreover, TfR-mediated iron delivery is thought to be essential to mitochondrial delivery through a “kiss-and-run” mechanism where the endosome carrying the (Fe^3+^)_2_-Tf-TfR complex can dock with the outer mitochondrial membrane to deliver iron through STEAP3 to Mfrn-1/2 for mitochondrial iron uptake [22]. Consistent with this premise, it has recently been shown that DMT1 coordinates endosomal–mitochondrial iron delivery to promote tumor metastasis [30]. Therefore, it can be purported from these data that gallium challenges the mitochondrial delivery process, resulting in impaired tumor growth; however, more extensive work will be required to fully elucidate this mechanism.

In addition to the observations regarding mitochondrial iron metabolism, this study also presents a novel concept, in that the generation of gallium-resistant cells through long-term, high-dose exposure results in high levels of cellular instability, resulting in robust radiosensitivity. These data further support the hypothesis that the mitochondrial iron depletion induced by gallium can limit the substrate (i.e., iron) required for the generation of critical [4Fe-4S]^2+^-containing DNA metabolic enzymes that preserve genomic integrity [14]. Moreover, disruption of the Fe-S machinery has been linked to genomic instability. For example, a loss of frataxin, the early-acting [2Fe-2S]^+^ biogenesis regulator associated with Friedreich’s ataxia, has been shown to exhibit elevated levels of nuclear damage in yeast and impair DNA base excision repair activation in both prokaryotic and eukaryotic cells [31,32]. There is a vast array of DNA metabolic enzymes that need [4Fe-4S]^2+^ clusters to function, including all DNA polymerases, DNA primase, DNA glycosylases involved in DNA damage repair (MUTYH/NTHL1), and DNA helicases [15]. This array of enzymes are essential for ensuring high-fidelity DNA replication and efficient DNA repair processing, which likely explains the significant impairments in cell growth associated with a single dose of Ga(NO_3_)_3_ and the generation of GaR1 and GaR2, which leads to the inhibition of mitochondrial uptake by knocking down Mfrn-1. Interestingly, the enhanced radiosensitivity of GaR1 and GaR2 only occurs at 2 Gy radiation. This is likely because at low-dose radiation, single-stranded DNA damage is the primary lesioning that occurs, while double-stranded breaks begin to occur at higher doses and the major DNA repair enzymes that utilize [4Fe-4S]^2+^ clusters are DNA glycosylases (e.g., MUTYH), which function in base excision repair to alleviate single-stranded breaks associated with oxidative damage [15,33,34]. The exacerbated cell killing in GaR1 and GaR2, when given 24 h following radiation to repair DNA damage, further corroborate this posit. The accumulation of damage, or impaired ability to repair radiation-induced damage by GaR1 and GaR2, is consistent with a study from 1982, which showed that fibroblasts isolated from individuals with Friedrech’s ataxia (i.e., a mutational loss of frataxin) exhibited the same phenotype as observed in this study and significant impairment of their ability to repair potentially lethal damage after radiation when compared to age-matched donor cells [35]. While these results are intriguing, they are not without limitation. The major limiting factor of this work is that it remains largely correlative, and further mechanistic studies are required to further elucidate the role(s) of Fe-S biogenesis in the regulation of cellular radiosensitivity. When taken together, these results suggest that disrupting mitochondrial iron metabolism with gallium can sensitize cells to low-dose radiation. 

## 4. Materials and Methods

### 4.1. Cell Culture

All glioma cells (U87, ATCC HTB-14 and U251, Millipore Sigma, Burlington, MA, USA) were cultured in DMEM-F12 media (15% FBS, 1% penicillin-strep, 1% Na-pyruvate, 1.5% HEPES, 0.1% insulin, and 0.02% fibroblast growth factor) and grown to 70–80% confluence at 21% O_2_ before experimentation. Cell lines were authenticated before use.

#### 4.1.1. Colony Formation Assay

For colony formation assays, cells were plated as single cells (500–2000 cells per dish) and allowed to grow for 7–12 days to allow for colony formation. The colonies were washed, fixed to the dish with 70% EtOH (stored at 4 °C), and stained with Coomassie blue. Colonies with > 50 cells were counted. Plating efficiency was calculated using the following formula:

(1) $$Plating\;efficiency\left(\%\right)=\left(\frac{\#\;colonies\;formed}{\#\;cells\;plated}\right)\times 100$$

Normalized survival fraction was calculated by normalizing the plating efficiency of the treated cells to the plating efficiency of the control group:

(2) $$Normalized\;survival\;fraction=\left(\frac{Plating\;efficiency\;(treatment)}{Plating\;efficiency\;(control)}\right)$$

#### 4.1.2. FtH Overexpression Model 

The FtH-pTRIPZ vectors were provided by the laboratory of Douglas Spitz and used as previously described [36,37]. To produce lentiviruses, TSA201 cells were used along with VSV-G and psPAX2 helper vectors (Addgene, Watertown, MA, USA). The viruses were collected from TSA201 cell cultures, centrifuged to remove cell debris, and filtered using 0.45 µm filters from the ZymoPURE^tm^ II Plasmid Midiprep Kit (Zymo Research, Irvine, CA, USA). The cells were plated and allowed to grow for 24 h, and then, the viruses were added to cells with 8 µg/mL of polybrene for a total of 48 h, with fresh viruses being added after 24 h. Following transduction, cells were selected with 2.5 µg/mL puromycin. The general population that survived the puromycin selection were then validated for overexpression by treating them with 1 µg mL^−1^ doxycycline hyclate (Fisher Bioreagents BP2653-5, Geel, Belgium) for 48 h. Low-density cell suspensions were then grown in a 96-well plate to form single-cell clones. Picked clones were also treated with 1 µg mL^−1^ doxycycline for 48 h to validate the overexpression (activity for GPx4 and Western blot analysis for FtH). 

### 4.2. Reagent Preparations

Ga(NO_3_)_3_ (Sigma #69365-72-6; St. Louis, MO, USA) was used from a 50 mM stock in H_2_O. Doxycycline was used at a concentration of 1 µg mL^−1^ from a 4 mg mL^−1^ stock in H_2_O.

### 4.3. Labile Iron Detection 

#### 4.3.1. Colorimetric Detection with Ferrozine 

Labile iron and total iron concentrations were assessed using a ferrozine-based colorimetric assay. Cells were homogenized in 1X RIPA lysis buffer (Sigma-Aldrich, St. Louis, MO, USA). The cells were centrifuged at maximum speed for 10 min to remove cell debris, and 100 µL of the supernatant was then diluted 1:1 in ferrozine buffer (5 mM ferrozine, 1.25 M ammonium acetate, 10 mM ascorbate) and centrifuged again at maximum speed for 10 min to remove any protein aggregates. This step is critical as the acidic nature of the buffer (pH ≈ 4–4.5) will result in protein aggregation, which can cause the samples to become cloudy and alter the absorbance profile, resulting in an experimental artifact. Thus, samples with remaining protein aggregates were removed from the analysis. The supernatant was then placed in a single well of a clear 96-well plate. Following dilution, the 96-well plate was evaluated for the formation of an Fe^2+^-ferrozine complex by monitoring the absorbance at 562 nm, and the Fe concentration was calculated using Beer’s Law:

(3) A562 (A.U.)=ε562∗[Fe]∗L

where A_562_ is the measured absorbance at 562 nm, ε562 is the molar extinction coefficient for an Fe^2+^-ferrozine complex = 27,900 M^−1^ cm^−1^, [Fe] is the calculated Fe concentration (M), and L is the pathlength for 200 µL of liquid ≈ 0.55 cm. The calculated [Fe] was normalized to the control cells. 

#### 4.3.2. Flow Cytometry Detection with Calcein-AM

Intracellular labile iron pool measures were performed using a Calcein-AM fluorescent dye. The cells were harvested by trypsinization. After cell harvesting, the cell pellets were washed in PBS and then resuspended in 500 nM Calcein-AM diluted in PBS. The samples were incubated for 15 min at 4% O_2_ (37 °C, 5% CO_2_). Following incubation, extracellular Calcein-AM was removed by washing with PBS, and the cells were resuspended in 1 mL PBS. Following incubation 10,000 cells were analyzed on an LSR II Flow Cytometer (BD Biosciences, Franklin Lakes, NJ, USA; λ_ex_ = 488 nm, λ_em_ = 515/20 nm). The labile iron pool was quantified using the following formula:

(4) $$relative\;LIP\;\left(A.U.\right)={\left(\frac{{MFI}_{treatment}}{{MFI}_{control}}\right)}^{-1}$$

An inverse normalization was performed to approximate the labile iron pool because calcein-AM functions as a “turn-off” probe. Mean fluorescence was quantified using FloJo software V.10.0.0.

### 4.4. Mito-FerroGreen Evaluation of Mitochondrial Iron

Mito-FerroGreen (Dojindo Laboratories, M489, Rockville, MD, USA) was used as a fluorescent probe for mitochondrial Fe^2+^. Following treatment, cells were washed with PBS, trypsinized, and pelleted by centrifugation. The cell pellets were washed once with PBS before resuspending with 5 µM Mito-FerroGreen and incubating for 30 min at 37 °C. After incubation, the cells were centrifuged, and residual stain was removed from the cell pellet. The cell pellets were washed with PBS three times and then resuspended in PBS for analysis. The cells were analyzed using the FITC channel of a Becton Dickinson LSR II flow cytometer (Thermo Fisher Scientific: Waltham, MA, USA).

### 4.5. Western Blotting

Cells were lysed in RIPA buffer (Sigma Aldrich, St. Louis, MO, USA), and total protein of the supernatant was quantified using a DC™ protein assay kit (Bio-Rad, Hercules, CA, USA). The proteins (20 μg each) were then separated by 4–15% SDS-PAGE electrophoresis (Bio-Rad) and transferred to 0.22 um pore size PVDF membranes (Bio-Rad, Hercules, CA, USA) for 1 h at 4 °C and 100 V. The membranes were then blocked with 5% non-fat dry milk in Tris-buffered saline/1% Tween-20 (TBS-T) for 1 h and incubated at 4 °C overnight with primary antibodies [Anti-transferrin (1:1000, Proteintech, Rosemont, IL, USA), anti-ferritin heavy chain (1:1000, Cell Signaling, Danvers, MA, USA), anti-Mitoferrin-1 (1:1000, Proteintech), and anti-β-actin (1:1000, Cell Signaling). The membranes were washed 3×, 5 min each with 1× TBST (Bio-Rad) and were incubated with either goat anti-mouse (1:5000) or anti-rabbit (1:5000) conjugated with HRP for 1 h at room temperature. After 10 min of washing the membranes with 1× TBS-T, the signals were developed with a chemiluminescent kit (Super Signal West Pico & Super Signal West Femto, Thermo Scientific, Tempe, AZ, USA) and exposed on an X-ray film (Research Products International, Mount Prospect, IL, USA). 

### 4.6. DNA Damage Analysis with Comet Assays

Single-strand DNA breaks were detected using alkaline comet assays. The R&D CometAssay Electrophoresis Starter Kit (#4250-050-ESK, Minneapolis, MN, USA) was used following the manufacturer’s instructions, with slight modifications as previously described [38]. Briefly, cells in agarose suspension were placed on 2-well comet slides and allowed to dry in the dark at 4 °C for 10 min. The slides were submerged in lysis buffer for 45 min at 4 °C and then incubated for 20 min in alkaline buffer at room temperature. Electrophoresis was carried out at 21 V for 30 min. The slides were then washed twice in water and once in 70% ethanol and were then left to dry at 37 °C for 15 min. 1X SYBR Gold (#S11494, ThermoFisher, Waltham, MA, USA) stain was used to stain the slides for 30 min at room temperature in the dark. The slides were briefly rinsed with water to remove excess stain and were then dried at 37 °C for 10 min. Fluorescent microscopy was used to capture images, and analysis was carried out using the autoanalyzer software CometScore 2.0.0.38 to obtain percent tail DNA (http://rexhoover.com/index.php?id=cometscore, access obtained on 21 December 2021).

### 4.7. ^67^Ga-Citrate Uptake

All studies involving ^67^Ga-citrate (purchased from Curium Pharma, 111 West Port Plaza Suite 800 Saint Louis, MO, USA) were carried out in a designated fume hood with adequate lead block shielding. All water was deionized and passed through Millipore water purification system until a resistivity of 18 MΩ·cm was achieved. For ^67^Ga-citrate cellular uptake, cells were seeded in a 6-well plate and were allowed to grow for 3–4 days to reach 60–80% confluence. The media was removed, and the attached cells were washed thrice with 1 mL ice-cold PBS, followed by the addition of 160–210 uCi (6–8 MBq) of ^67^Ga-citrate in 3–4 mL media. The cells were incubated at 37 °C for 3–4 h followed by washing with ice-cold PBS (3–5 mL) to remove unbound ^67^Ga-citrate. The cells were then detached from the plate with a scrapper into 2 mL Eppendorf vials (175 Freshwater Blvd Enfield, CT, USA) and were centrifuged to allow the cells to settle at the bottom of the vial, and the remaining PBS was removed. All experiments were performed in triplicate. Radioactivity taken up by the cells was countered using a gamma counter (Perkin-Elmer, Wizard2 2480, Waltham, MA, USA), and radioactivity in the samples was decay-corrected using the gallium-67 protocol supplied by the manufacturer.

### 4.8. Statistical Methods

Experiments were performed in triplicate with α = 0.05 being used as a threshold to determine statistical significance. Statistical analysis was performed using GraphPad Prism software V9.5.1. 

## 5. Conclusions

This study shows that gallium can induce a mitochondrial iron depletion phenotype that is associated with DNA damage. Moreover, repeated high doses of gallium generate a stable, mitochondrial iron depletion phenotype that is generally unstable and exhibits significant sensitivity to ionizing radiation. The sensitivity to ionizing radiation may be mechanistically linked to an Fe-S substrate limitation that impairs the generation of [4Fe-4S]^2+^ clusters required for high-fidelity DNA repair. Taken together, these data support the future use of gallium as a radiosensitizer in cancer therapy, which warrants further investigation from both a mechanistic and translational science perspective.

## Data Availability

Data is contained within the article or Supplementary Material.

## Supplementary Materials

The following supporting information can be downloaded at https://www.mdpi.com/article/10.3390/ijms251810047/s1.
