# Supplementary material for: Gallium Uncouples Iron Metabolism to Enhance Glioblastoma Radiosensitivity
Source: Int J Mol Sci. 2024 Sep 18;25(18):10047. doi: 10.3390/ijms251810047 (PMC11432413; doi:10.3390/ijms251810047)
Supplement: Supplementary file 1 [file ijms-25-10047-s001.zip › ijms-3183118-supplementary.pdf]

Supplemental Information for *International Journal of Molecular Sciences*

Gallium Uncouples Iron Metabolism to Enhance Glioblastoma Radiosensitivity

SStephenson B. Owusu [1], Amira Zaher [1], Stephen Ahenkorah [2], Darpah N. Pandya[2], Thaddeus J. Wadas [2] and Michael S. Petronek [1,*]

1 Department of Radiation Oncology, Division of Free Radical and Radiation Biology, The University of Iowa, Iowa City, IA 52242, USA

2 Department of Radiology, The University of Iowa, Iowa City, IA 52242, USA

Page 2: Supplemental Figure 1. Effects of $Ga(NO_3)_3$ on labile iron.

Page 3: Supplemental Figure 2. $Ga(NO_3)_3$ toxicity is not mitigated by ferritin expression.

Page 4: Supplemental Figure 3. $Ga(NO_3)_3$ toxicity is not affected by holo-transferrin supplementation.

Page 5: Supplemental Figure 4. Ferrous ammonium sulfate enriches mitochondrial iron.

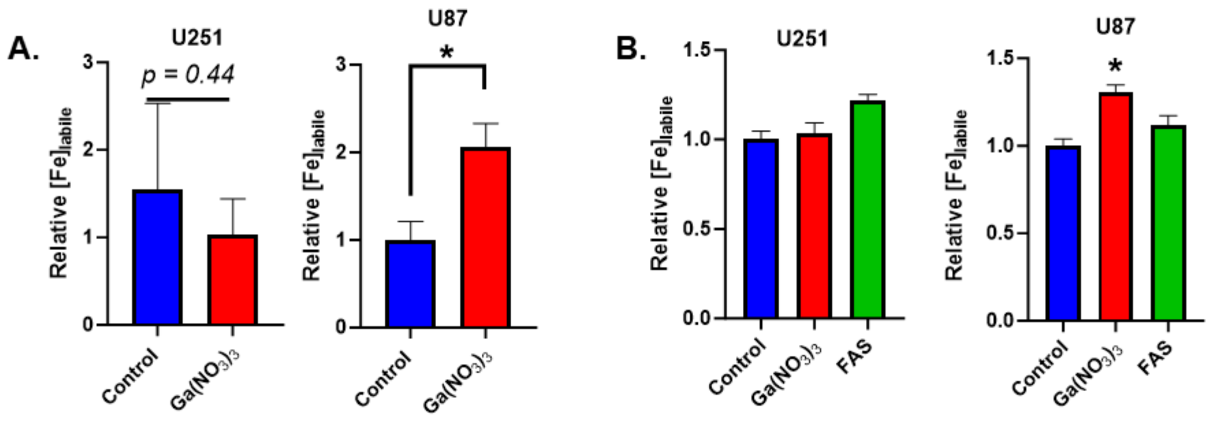

Supplemental Figure S1. Effects of $Ga(NO_3)_3$ on labile iron. A. Labile iron pool (LIP) measures in U251 and U87 glioblastoma cells following a 48 h treatment with 500 µM $Ga(NO_3)_3$ determined colormetrically using ferrozine buffer (A_{562}, ε_{562} = 27,900 L mol^{-1} cm^{-1}). Error bars represent mean ± SD of 3 biological replicates with *$p<0.05$ using a Welch's T-test. **B.** Chelatable iron in U251 and U87 glioblastoma cells following a 6 h treatment with 500 µM $Ga(NO_3)_3$ or ferrous ammonium sulfate determined using calcein-AM. Error bars represent mean ± SEM of 3 biological replicates with *$p<0.05$ using a one way ANOVA-test. For panels B and C, chelatable Fe concentrations were normalized to the untreated control.

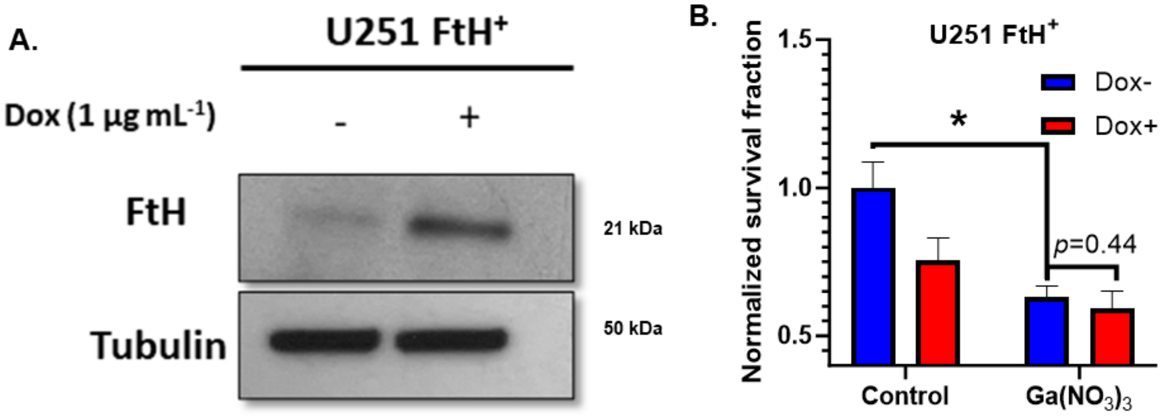

Supplemental Figure S2. $Ga(NO_3)_3$ toxicity is not mitigated by ferritin expression. A. Western blot analysis of U251 cells overexpressing FtH (48 h, 1 µg mL^{-1} doxycycline). These genetically modified cells were used to test the hypothesis that $Ga(NO_3)_3$ toxicity is due to the displacement of Fe from proteins, which may serve as a labile, redox-active Fe source capable of catalyzing reactive oxygen species. If this were the case, FtH would serve as an Fe storage reserve to absorb the displaced Fe, ultimately mitigating the $Ga(NO_3)_3$ toxicity. **B.** Clonogenic survival analysis of FtH overexpressing U251 cells following a 48 h treatment of 500 µM $Ga(NO_3)_3$. FtH expression was induced using a 24 h pretreatment of doxycycline (1 µg mL^{-1}) followed by a 24 h treatment of 500 µM $Ga(NO_3)_3$. Error bars represent mean ±SEM of 3 biological replicates with *$p<0.05$ using a one way ANOVA-test with a post-hoc Tukey's test for multiple comparisons.

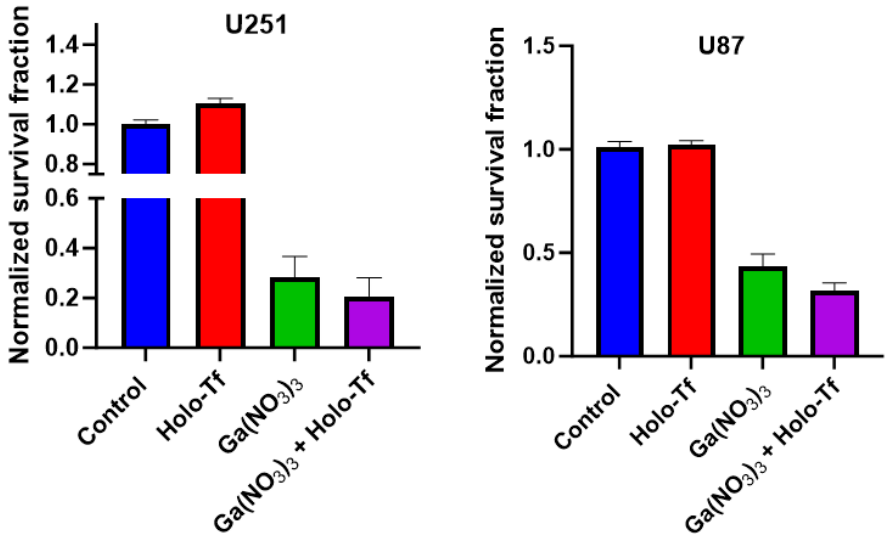

Supplemental Figure S3. $Ga(NO_3)_3$ toxicity is not affected by holo-transferrin supplementation. Clonogenic survival analysis of U251 and U87 cells following a 48 h treatment of 500 µM $Ga(NO_3)_3$ ±100 µg mL^{-1} supplement of holo-transferrin (holo-Tf). Error bars represent mean ±SEM of 3 biological replicates with *$p<0.05$ using a one-way ANOVA-test with a post-hoc Tukey's test for multiple comparisons.

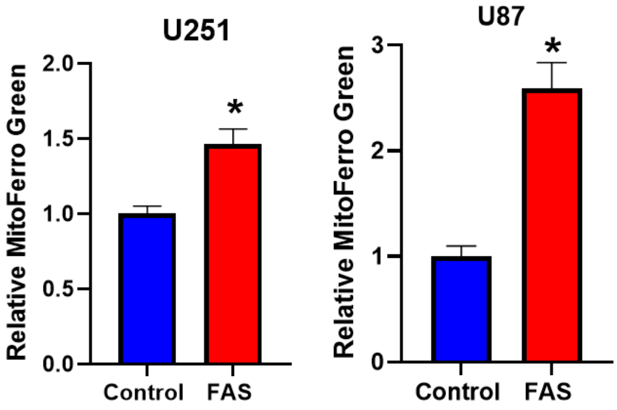

Supplemental Figure S4. Ferrous ammonium sulfate enriches mitochondrial iron. MitoFerroGreen staining of U251 and U87 cells following a 3 h treatment of 500 µM FAS Error bars represent mean ±SEM of 3 biological replicates with $*p<0.05$ using a Welch's T-test.
